# A prospective registry-based cohort study of the diagnosis and management of acute leukaemia in pregnancy: Study protocol

**DOI:** 10.1371/journal.pone.0263195

**Published:** 2022-02-07

**Authors:** Matthew Northgraves, David Allsup, Judith Cohen, Chao Huang, John Turgoose, Sahra Ali

**Affiliations:** 1 Hull Health Trials Unit, University of Hull, Hull, United Kingdom; 2 Centre for Atherothrombosis and Metabolic Disease, Hull York Medical School, Hull, United Kingdom; 3 Institute for Clinical and Applied Health Research (ICAHR), University of Hull, Hull, United Kingdom; 4 King’s College Hospital NHS Foundation Trust, Denmark Hill, London, United Kingdom; UNITED KINGDOM

## Abstract

**Background:**

Acute leukaemias (AL) are aggressive but potentially curable blood cancers that can potentially affect women of childbearing age. When a pregnancy is complicated by a diagnosis of AL, clinicians face a complex dilemma: to balance risking the mother’s survival through delayed AL treatment, against the potential harm to the foetus through exposure to anti-cancer drugs. Up until now, all guidance and advice regarding the management of AL in pregnancy, have been based on expert opinion and small case studies. There is a pressing need for more studies in the subject to address this evidence gap.

**Methods and analysis:**

This study is a registry-based observational cohort study which aims to monitor and record the treatment outcomes of patients diagnosed with AL during pregnancy. Additionally, the study aims to assess pregnancy outcomes in patients who become pregnant following successful treatment. Prospective and historical cases from August 2009 onwards will be identified from AL treating haematology units within the UK. Details of diagnosis, AL treatment delivered, antenatal and postnatal outcomes for mother and neonate will be collected. This study will establish a new research database for Leukaemia in Pregnancy.

**Trial registration:**

The study was registered on Clinicaltrials.gov (NCT04182074) on the 2^nd^ December 2019.

## Introduction

Acute leukaemias (AL) are rare, life-threatening malignancies with an incidence of 1 per 100,000 in women under the age of 50 [1] therefore potentially affecting women of childbearing age. Although estimated to occur in only 1 in 75,000 to 100,000 pregnancies [2], AL remains one of the most frequent malignant diseases observed during pregnancy [3]. AL has been described during the antenatal and postnatal periods [3, 4]. When diagnosed during pregnancy, the treating multidisciplinary team are presented with a series of unique clinical dilemmas. The risk to the mother through delaying AL treatment needs to be balanced against the potential harm chemotherapeutic drugs could have on the developing foetus. Evidence suggests that successful treatment of AL during the second and third trimesters with the foetus in utero is feasible [4] with AL complete remission rates of up to 80% after standard chemotherapy [3, 5]. Additionally, for the AL subtype acute promyelocytic leukaemia (APL), all-trans-retinoic acid (ATRA) therapy in combination with conventional chemotherapy has been successfully delivered during the antenatal period [6–8]. Treating AL to achieve disease remission at delivery has been associated with decreased maternal and foetal morbidity and mortality compared with untreated or partially treated leukaemia [9]. The current British Committee for Standards in Haematology (BCSH) guidelines published in 2015 [9] make specific recommendations on diagnostic and management issues associated with acute myeloid leukaemia (AML) during pregnancy. Although of great value, these were informed by only limited evidence, with many of the recommendations derived from expert opinion. Furthermore, there is a dearth of evidence pertaining to the treatment of acute lymphoblastic leukaemia (ALL) during pregnancy which has so far precluded the establishment of firm recommendations [10]. With the prevalence of cancer during pregnancy expected to rise due to an increase in average age at pregnancy [11], there is a real and urgent need to establish firm recommendations on the safe and effective therapeutic management of pregnancy-related AL.

For female cancer survivors of reproductive age the preservation of fertility and the potential for future parenthood are important quality of life issues [12]. Unfortunately, the various treatment options available for AL can adversely affect fertility [12–15]. Due to the nature of AL, lower intensity chemotherapy regimens cannot be considered as these would comprise clinical outcomes. Additionally, treatment must be started immediately after diagnosis due to the rapid clinical course of AL. These factors preclude pre-treatment options for pregnant women with AL for the preservation of fertility, such as oocyte retrieval and storage. Despite this, there have been reports of successful pregnancies following treatment of AML, APL and ALL, both with and without the use of assisted reproductive technology [16], and it appears that the likelihood of ovarian failure (and the chances of subsequent recovery of ovarian function) depend on a variety of factors including patient age, type of cytotoxic agent used, and the cumulative chemotherapy dose [14].

There is no standard approach for the treatment of AL during pregnancy and the effects of such therapy on fertility post treatment are not fully understood. Current therapeutic approaches to pregnancy related AL may be highly variable, and differ from those used in non-pregnant patients. Given the rarity of pregnancy-related AL there is a paucity of evidence concerning diagnosis and management that cannot be addressed either practically or ethically by randomised clinical trials. There is also a crucial need to gather data on pregnancy-related outcomes amongst women who previously received treatment for AL. Evidence is currently limited to individual case reports, small retrospective case series and reviews of a small number of patients. Whilst there are UK national datasets [17, 18] specific to cancer collecting data regarding aspects such as diagnosis, treatment response and survival in leukaemia, none specifically focus on pregnancy outcomes in these patients. The UK Obstetric Surveillance System (UKOSS), is a unique national reporting system for collecting prospective cases of uncommon disorders of pregnancy [19], but AL is one of the conditions that has never been reported in this dataset. The international cancer in pregnancy registry (INCIP) [20] is ongoing with the majority of cases registered from Belgium, the Netherlands, Italy, and the USA. There have been no publications from INCIP regarding the treatment of AL in pregnancy. Such data will inform future treatment recommendations for the initial care of women of reproductive age diagnosed with AL, as well as the management of any future pregnancies.

## Aims and objectives

The leukaemia in pregnancy research database aims to capture both retrospective and prospective epidemiological, management and outcome data for women who either received a diagnosis of AL or high-risk MDS in pregnancy or subsequently conceived following AL treatment.

The main objectives are to ascertain:

### 1. Maternal and neonatal outcomes

The maternal and neonatal clinical outcomes following treatment for AL or high-risk MDS during or prior to pregnancy.

### 2. Chemotherapuetic management of AL and MDS during pregnancy

The current management approach for women diagnosed with AL or high-risk MDS during pregnancy or who become pregnant after receiving therapy will be documented so changes over time can be observed.

### 3. Treatment safety

The safety of medications used to treat AL and MDS by identification of any maternal or neonatal toxicities.

### *Secondary objective*:

This study will establish an ongoing Leukaemia in Pregnancy research database to provide a source of data for future research and analysis.

## Methods and analysis

### Study design

This study is a registry-based observational cohort study which will establish the Leukaemia in Pregnancy research database. Both prospective and historical cases from August 2009 onwards will be identified and data entered using routinely collected information from NHS hospital records about AL diagnosis and treatment, pregnancy, delivery and the outcomes of childbirth. Data will be collected up to four years post-delivery in order to accurately document the subsequent outcomes of the mother and obtain details of any further pregnancies within the follow-up period.

### Study setting

UK hospitals with high intensity haematology units treating AML and ALL. The study team will attempt to engage with all UK centres for case identification.

### Eligibility criteria

Eligible cases are defined as women have had a diagnosis of AL or high-risk MDS during pregnancy, or who have later conceived after previously being diagnosed and receiving treatment for either AL or high-risk MDS.

### Cases identification and classification

Two types of cases will be collected within the leukaemia in pregnancy research database:

1. Prospective cases where the woman is currently pregnant whilst receiving treatment or having previously had treatment.
2. Historical cases since August 2009, where a patient has had a previous pregnancy and either received treatment during that pregnancy or became pregnant having previously received treatment.

Case identification will be completed by clinical staff at participating sites via a retrospective review of medical notes, clinic appointment lists and hospital databases of patient-level diagnostic and mortality data.

#### Requirements for consent

**Fig 1:**
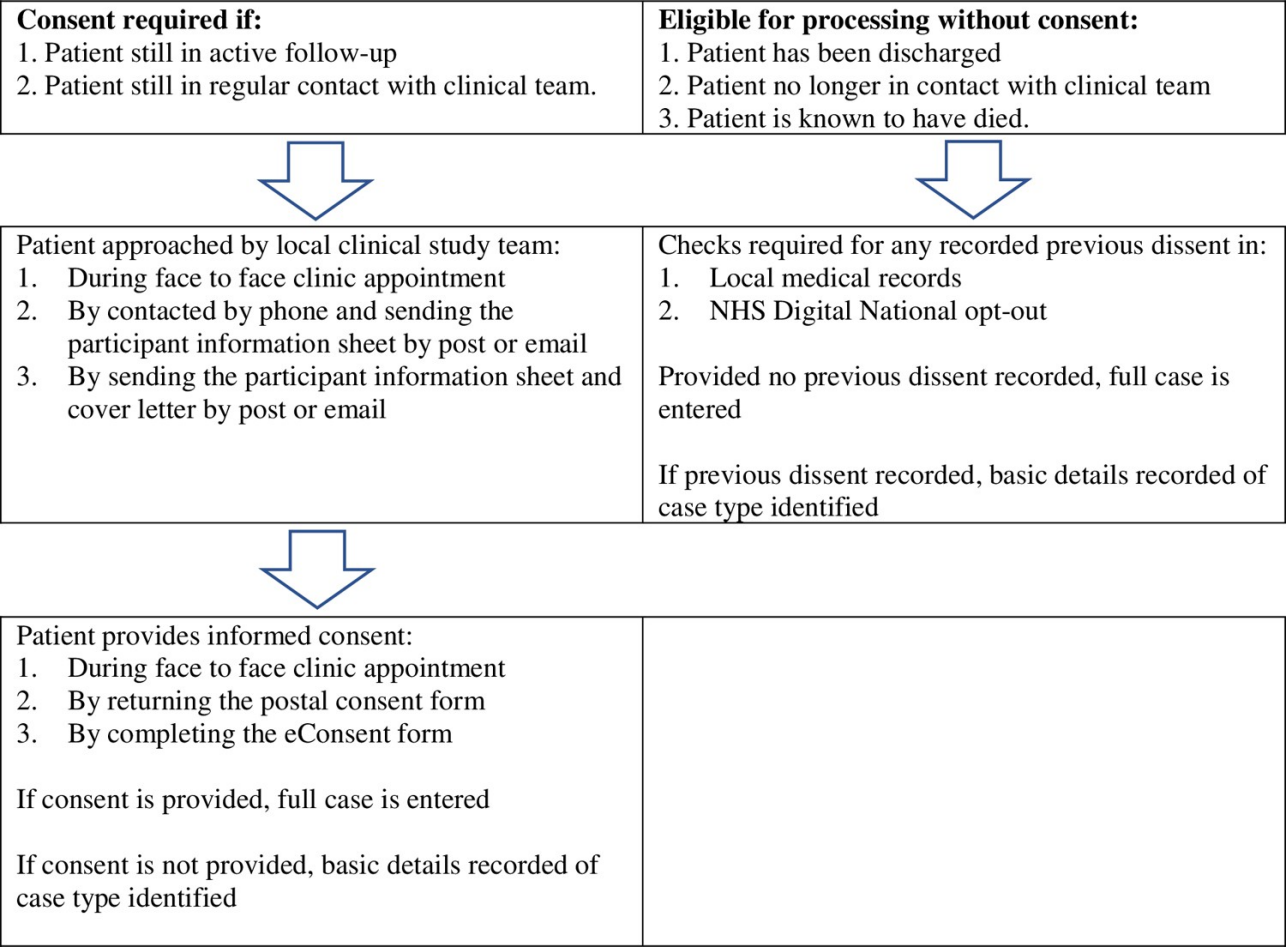
Procedure for processing cases with and without consent.

During the set-up of the study the requirements for obtaining consent to collect routinely collected data, and the acceptability of different approaches was explored at a patient and public involvement meeting. There is the potential to cause unnecessary emotional distress by contacting patients or their families as in some instances as the patient and/or their babies will not have survived. It was agreed that written informed consent taken by the clinical care or site research team should be sought from all identified prospective cases and any historical cases where the patient is still in active follow up and/or contact with the clinical care team. Consent can be face-to-face, postal or eConsent. However, it was not considered reasonable or practicable to contact patients who are either no longer in active clinical follow-up or in contact with the clinical care team, or are known to have died. It is possible to collect data in these circumstances with approval from the Confidentiality Advisory Group in England and Wales [21] and the Patient Benefit and Privacy Panel in Scotland [22] provided checks for previous dissent against the use of their data for the purposes of research are made. As no directly identifiable data is being collected, this is done according to the participating sites own local policy but as a minimum must include a check of local records for evidence of any historic dissent. In addition, for cases in England, processed under the CAG approval, checks against registration to the NHS Digital National opt-out [23] is required. The procedure for processing cases with and without consent is outlined in Fig 1.

### Data collection

Once an eligible case has been identified, the delegated local study team member will enroll the case into the leukaemia in pregnancy research database. An initial screening form confirms patient consent and dissent status. Depending on case type, providing the patient has either given informed consent or no previous dissent has been recorded, the case is fully enrolled into the database generating a unique patient ID. To enable completion of a CONSORT diagram, basic information on cases deemed eligible but not enrolled will be captured including the reason for non-enrolment. Sites will maintain a participant enrolment log linking the pseudonymised patient study number in the database to patient identifiable information at site, allowing the participating sites to obtain further information from clinical records and to complete follow-up data collection forms.

Once a case record is created, electronic case report forms are generated in the database for the relevant time points according to the patient group. The schedule of data collection is outlined in Table 1 with a detailed list of the variables being collected available in S1 File.

**Table 1:** Schedule of events.

AL diagnosis during Pregnancy		Pregnancy following AL treatment	
Event*	Details collected	Event*	Details collected
Diagnosis event	Diagnosis Full blood count Biochemical panel Clotting data Bone marrow aspirate	Diagnosis and treatment event	Diagnosis Treatment Post-treatment
Pregnancy event	Pregnancy Treatment	Pregnancy event	Pregnancy
Delivery and Outcome event	Delivery Outcomes for mother Outcomes for infant Mother and Infants Full Blood Count Biochemical Panel	Delivery and Outcome event	Delivery Outcomes for mother Outcomes for infant Mother and Infants Full Blood Count Biochemical Panel
2 Year follow-up event	Follow-up Further pregnancies	2 Year follow-up event	Follow-up Further pregnancies
4 Year follow-up event	Follow-up Further pregnancies**	4 Year follow-up event	Follow-up Further pregnancies**

* Events are the points in the patient journey when the data is collected; If there are any further pregnancies the details in the ‘Pregnancy’ and ‘Delivery and Outcome’ events are repeated.

For the previous AL case type, the index case is the date of the first pregnancy following treatment that remains viable over 24 weeks.

Follow-up timepoints are 2 and 4 years post-delivery and include information regarding remission status and details of any further pregnancies. If a woman is pregnant at time of the 4 year post-delivery follow-up, information about the pregnancy and delivery will be collected up to the end of the pregnancy.

### Data management

The leukaemia in pregnancy research database is being hosted and managed by the Hull Health Trials Unit (HHTU) using the secure online data capture system REDCap Cloud®. Automated checks are built into the database with a dedicated data manager from the HHTU overseeing and monitoring the conduct of the database. For current cases and historical cases requiring consent, the patient name and signature will be captured as part of the consent process with either scanned copies of the written informed consent uploaded into RCC or eConsent gained via Docusign®. Consent forms will only be visible to designated staff at sites and the central HHTU team.

At the end of the initial study period and annually thereafter data will be exported from the data collection database allowing the HHTU based database statistician to make survival calculations. For any patients who have reached the 4-year follow-up time point, data from the data collection database will be used to create three new variables: 1) Child age in weeks at death (up to 30 days post-delivery) and 2) Age (years/months) of mother at death (collected on outcome, 2 year follow-up or 4 year follow-up as applicable; 3. If still alive, Age at 4-year follow-up. These will replace child’s date of birth and date of deaths (mother and child). All other dates (e.g. treatment dates, ultrasound scans) for the patient will also be altered once they have reached the 4-year follow-up time point according to a random date off set. This dataset will be added to a second ‘long-term’ database for which all cases will be recorded as a single site for the whole of the UK. Once the data is in the ‘long-term’ database, the original data will be deleted from the data collection database making it anonymised. At this point it will be no longer be possible to identify and therefore remove the data for that case. Other researcher will be able to request access to the anonymised patient-level data held within the leukaemia in pregnancy research database. A data management panel, will review and approve requests.

### Sample size and data analysis

This is an observational cohort study with the main aim of summarising management regimens and complications for AL in pregnancy. The study was not powered for formal statistical comparison or evaluation, but aims to establish the largest dataset of cases of AL in pregnancy to date. Considering that the number of prospective cases expected in the UK is approximately 10 cases per year, the target number of cases for this study is 30–50 cases. Beyond the duration of this study, the aim is to continue to add cases to enable further future analyses.

The reporting guideline of observational studies (STROBE) [24] will be followed for analysis and reporting of this study. The statistical analysis will be mostly descriptive, and the resulting report will detail current and past management regimens and complications for AL in pregnancy. Mean (SD) or median (IOR) will be reported for continuous data and raw count (number, %) will be reported for nominal data. The outcomes of the mother and babies during or prior to pregnancy, the current management practices for patients with AL or high-risk MDS during pregnancy or who become pregnant after receiving therapy, and the safety of any medication used to treat the mothers will be summarised. We will also report the cases before and after the year 2015 to examine the impact of the introduction of the BCSH guidelines [9]. Providing a satisfactory response rate is achieved, incidence data will be included, calculated with 95% confidence intervals.

### Patient and public involvement

A patient and public involvement session was held with the Trans-Humber Consumer Research Panel to inform in the design of the leukaemia in pregnancy research database. In particular, issues around the consent procedure, the appropriateness of processing certain cases without consent and future plans for data requests were discussed.

### Ethics and dissemination

#### Regulatory approvals and trial oversight

NHS research database REC approval was granted on the 2^nd^ August 2019 by the South Central -Oxford B Research Ethics Committee for the Leukaemia in Pregnancy Research database (19/SC/0356). As a research database, HRA approval was not required [25]. Research ethical approval for the Leukaemia in Pregnancy Study was given by Hull York Medical School Research Ethics Committee on the 16^th^ June 2019 (19 23). For the processing of certain cases without consent, support from the Confidentiality Advisory Group (CAG) for England and Wales was obtained on the 18^th^ September 2019 (19/CAG/0136) and from the Patient Benefit and Privacy Panel for Scotland on the 13^th^ January 2021 (14920–0137). The study sponsor is the University of Hull and the database is managed by the HHTU. A Trial Management Group (TMG), comprised of the Chief Investigator, the local principle investigator and the HHTU staff oversee the study implementation and progress. An extended TMG, including other representative authors of the BCSH guidelines [9] will assist with the establishment of the database and interpretation of the data collected.

#### Amendments

On the basis of certain conditions relating to dissent procedures that were requested by the CAG and the addition of collecting information regarding any further pregnancies, REC Substantial amendment (SA) 1 was approved on the 9^th^ October 2019. The addition of further pregnancies required an amendment to the CAG support which was gained on the 10^th^ December 2019. Due the COVID-19 pandemic SA2 adding the option for postal and e-consent was approved on the 18^th^ May 2020.

### Dissemination

The study results will be disseminated through presentations, conferences and peer-reviewed journals according to the LIPS publication and dissemination policy. Clinicians who contribute at least one case and fulfil in ICMJE (International Committee of Medical Journal Editors) recommendations will be offered authorship on the main results paper.

### Protocol version

The current protocol version is V2.1 (16.06.2020) which was implemented prior to the first case being entered on the 23^rd^ July 2020.

## Discussion

Despite the presence of a number of ongoing cancer and leukaemia registries [17–19, 26], only one [20], specifically captures information relating to patients either receiving treatment during their pregnancy or the effects of previous treatment on subsequent pregnancies. This dataset however contains minimal UK data. Through targeting all high intensity haematology units in the UK as recruitment centres, the leukaemia in pregnancy study will look to establish a comprehensive UK dataset to better identify past and current treatment regimens and their effects on maternal and neonatal outcomes. This will enable us to have evidence for the use of some new treatments including targeted therapy in some patients with specific mutations for whom these treatments are now available. We can compare and study neonatal toxicity of these drugs, if any. The dataset will help clinicians to understand how best to treat refractory cases, with more recent cases potentially avoiding some aspects of a regimen with potential fetal toxicity without compromising outcomes for the mother. The initial study and research database will serve not only as a source of information to support care decisions for pregnancies during AL treatment, but also as a late effects study to report on the feasibility of viable pregnancies post-treatment.

## Supporting information

S1 File List of data variables. (DOCX)

S1 Protocol (PDF)

S1 Checklist (DOC)

## References

[1] Haematological Malignancy Research Network. Incidence statistics: Overall incidence—Total selected diagnoses 2021 [Available from: https://hmrn.org/statistics/incidence.

[2] Pavlidis NA. Coexistence of Pregnancy and Malignancy. The Oncologist. 2002;7(4):279–87. 12185292

[3] Reynoso EE, Shepherd FA, Messner HA, Farquharson HA, Garvey MB, Baker MA. Acute leukemia during pregnancy: the Toronto Leukemia Study Group experience with long-term follow-up of children exposed in utero to chemotherapeutic agents. J Clin Oncol. 1987;5(7):1098–106. doi: 10.1200/JCO.1987.5.7.1098 3474357

[4] Chelghoum Y, Vey N, Raffoux E, Huguet F, Pigneux A, Witz B, et al. Acute leukemia during pregnancy: a report on 37 patients and a review of the literature. Cancer. 2005;104(1):110–7. doi: 10.1002/cncr.21137 15912518

[5] Döhner H, Estey EH, Amadori S, Appelbaum FR, Büchner T, Burnett AK, et al. Diagnosis and management of acute myeloid leukemia in adults: recommendations from an international expert panel, on behalf of the European LeukemiaNet. Blood. 2010;115(3):453–74. doi: 10.1182/blood-2009-07-235358 19880497

[6] Carradice D, Austin N, Bayston K, Ganly PS. Successful treatment of acute promyelocytic leukaemia during pregnancy. Clin Lab Haematol. 2002;24(5):307–11. doi: 10.1046/j.1365-2257.2002.00459.x 12358893

[7] Agarwal K, Patel M, Agarwal V. A Complicated Case of Acute Promyelocytic Leukemia in the Second Trimester of Pregnancy Successfully Treated with All-trans-Retinoic Acid. Case Rep Hematol. 2015;2015:634252. doi: 10.1155/2015/634252 25821608 PMC4363600

[8] Sanz MA, Montesinos P, Casale MF, Díaz-Mediavilla J, Jiménez S, Fernández I, et al. Maternal and fetal outcomes in pregnant women with acute promyelocytic leukemia. Ann Hematol. 2015;94(8):1357–61. doi: 10.1007/s00277-015-2372-5 25911134

[9] Ali S, Jones GL, Culligan DJ, Marsden PJ, Russell N, Embleton ND, et al. Guidelines for the diagnosis and management of acute myeloid leukaemia in pregnancy. Br J Haematol. 2015;170(4):487–95. doi: 10.1111/bjh.13554 26081614

[10] Shapira T, Pereg D, Lishner M. How I treat acute and chronic leukemia in pregnancy. Blood Rev. 2008;22(5):247–59. doi: 10.1016/j.blre.2008.03.006 18472198

[11] Brenner B, Avivi I, Lishner M. Haematological cancers in pregnancy. Lancet. 2012;379(9815):580–7. doi: 10.1016/S0140-6736(11)61348-2 22325663

[12] Stensheim H, Cvancarova M, Møller B, Fosså SD. Pregnancy after adolescent and adult cancer: a population-based matched cohort study. Int J Cancer. 2011;129(5):1225–36. doi: 10.1002/ijc.26045 21387311

[13] Das M, Shehata F, Son WY, Tulandi T, Holzer H. Ovarian reserve and response to IVF and in vitro maturation treatment following chemotherapy. Hum Reprod. 2012;27(8):2509–14. doi: 10.1093/humrep/des143 22617122

[14] Naessén S, Bergström I, Ljungman P, Landgren BM. Long-term follow-up of bone density, general and reproductive health in female survivors after treatment for haematological malignancies. Eur J Haematol. 2014;93(2):137–42. doi: 10.1111/ejh.12317 24649942

[15] Carter A, Robison LL, Francisco L, Smith D, Grant M, Baker KS, et al. Prevalence of conception and pregnancy outcomes after hematopoietic cell transplantation: report from the Bone Marrow Transplant Survivor Study. Bone Marrow Transplant. 2006;37(11):1023–9. doi: 10.1038/sj.bmt.1705364 16604098

[16] Wang WS, Tzeng CH, Hsieh RK, Chiou TJ, Liu JH, Yen CC, et al. Successful pregnancy following very high-dose total body irradiation (1575 cGy) and bone marrow transplantation in a woman with acute myeloid leukemia. Bone Marrow Transplant. 1998;21(4):415–7. doi: 10.1038/sj.bmt.1701106 9509978

[17] Henson KE, Elliss-Brookes L, Coupland VH, Payne E, Vernon S, Rous B, et al. Data Resource Profile: National Cancer Registration Dataset in England. International Journal of Epidemiology. 2019;49(1):16–h. 10.1093/ije/dyz076 PMC7124503 31120104

[18] Bright CJ, Lawton S, Benson S, Bomb M, Dodwell D, Henson KE, et al. Data Resource Profile: The Systemic Anti-Cancer Therapy (SACT) dataset. International Journal of Epidemiology. 2019;49(1):15–l. 10.1093/ije/dyz137 PMC7426029 31340008

[19] Juliusson G, Lazarevic V, Hörstedt AS, Hagberg O, Höglund M. Acute myeloid leukemia in the real world: why population-based registries are needed. Blood. 2012;119(17):3890–9. doi: 10.1182/blood-2011-12-379008 22383796 PMC3358248

[20] Maggen C, Wolters V, Cardonick E, Fumagalli M, Halaska MJ, Lok CAR, et al. Pregnancy and Cancer: the INCIP Project. Curr Oncol Rep. 2020;22(2):17. doi: 10.1007/s11912-020-0862-7 32025953 PMC7002463

[21] Health Research Authority. Confidentiality Advisory Group 2021 [Available from: https://www.hra.nhs.uk/approvals-amendments/what-approvals-do-i-need/confidentiality-advisory-group/.

[22] Public Health Scotland. Public Benefit and Privacy Panel for Health and Social Care-HSC-PBPP 2021 [Available from: https://www.informationgovernance.scot.nhs.uk/pbpphsc/.

[23] NHS Digital. National data opt-out 2021 [Available from: https://digital.nhs.uk/services/national-data-opt-out.

[24] von Elm E, Altman DG, Egger M, Pocock SJ, Gøtzsche PC, Vandenbroucke JP. Strengthening the Reporting of Observational Studies in Epidemiology (STROBE) statement: guidelines for reporting observational studies. Bmj. 2007;335(7624):806–8. doi: 10.1136/bmj.39335.541782.AD 17947786 PMC2034723

[25] Health Research Authority. Research tissue banks and research databases 2021 [updated 7th July 2021. Available from: https://www.hra.nhs.uk/planning-and-improving-research/policies-standards-legislation/research-tissue-banks-and-research-databases/.

[26] Ostgård LS, Nørgaard JM, Severinsen MT, Sengeløv H, Friis L, Jensen MK, et al. Data quality in the Danish National Acute Leukemia Registry: a hematological data resource. Clin Epidemiol. 2013;5:335–44. doi: 10.2147/CLEP.S48411 24039451 PMC3770716

