# Supplementary material for: A prospective registry-based cohort study of the diagnosis and management of acute leukaemia in pregnancy: Study protocol
Source: PLoS One. 2022 Feb 7;17(2):e0263195. doi: 10.1371/journal.pone.0263195 (PMC8820608; doi:10.1371/journal.pone.0263195)
Supplement: S1 File — (DOCX) [file pone.0263195.s001.docx]

**Leukaemia in Pregnancy registry Study (LIPS) data variables**

**Screening form**

- Case type (Historical/Current)
- If historic - In active follow-up where obtaining consent is practicable (Yes/No)

*If current or ‘Yes’ obtaining consent is practicable*

- - Consented (Yes/No)
  - Consent Date

*If no, (patients no longer in active follow-up processed without consent)*

- Has the patient been checked for any previous dissent to use of their data in research? Y/N
- Any previous dissent recorded?

**Subject details eCRF**

- Patient group
- Mothers Year of Birth
- Smoking status (Never/Current/Gave up prior to pregnancy/Gave up during pregnancy)

**Diagnosis eCRF**

*Diagnosis (All)*

- AL date of diagnosis
- Subtype of AL (according to WHO classification)
- MDS diagnosis date and classification
- IPSS score

*Additional information (Current AL only)*

- Did the woman have pre-existing medical conditions
  - Details and medications
- Symptoms at presentation

**Pregnancy eCRF**

*Pregnancy (Previous AL)* **(Collected for Index pregnancy only)**

- Is this the first pregnancy over 24 weeks after treatment? Yes/No
- Number of pregnancies less than 24 weeks since treatment
  - Number of terminations [number box]

*This pregnancy (All)* **(Collected for Index pregnancy and at 2 and 4 year follow-ups if applicable)**

- Estimated delivery date by dating scan at 12-14 weeks (Previously ‘Estimated final delivery date’)
- Was this a multiple pregnancy?
  - If yes, number of foetuses
- Was conception natural? Yes/No (If no, method of conception)
- Was an echocardiogram performed during pregnancy? Yes/No
  - If yes, Dates of scan
  - Results (Heart failure/Heart enlargement (Cardiomegaly)/Normal)

*This pregnancy (Current AL)* **(Collected for Index pregnancy)**

- Were ultrasound scans performed during treatment? [Previously ‘Was an ultrasound performed during treatment’] Yes/No [Previously ‘Was an ultrasound performed during treatment’]
  - If yes, date of scans, gestation stage (weeks), ultrasound result.
- Did the woman receive any blood transfusions during pregnancy? Yes/No
  - If yes, date/time of transfusion, number of units, indication)

APL patients

- Coagulation support details during pregnancy

*Complications* **(Collected for Index pregnancy and at 2 and 4 year follow-ups if applicable)**

- Were there any complications during the pregnancy? Yes/No
  - Specify complication (Pre-eclampsia, Gestational hypertension, Postpartum haemorrhage, Other)

**Delivery eCRF (Collected for Index pregnancy and at 2 and 4 year follow-ups if applicable)**

*Delivery*

- Did this woman have a miscarriage? Yes/No
  - If yes, date
- Did this woman have a termination? Yes/No
  - If yes, Week of gestation at termination
- What was the status of AL during pregnancy? Relapse/No change
- Was the woman in remission at the time of delivery?
- Was any molecular monitoring performed prior to delivery?
  - If yes, result
- Date of delivery
- Mode of delivery (Spontaneous vaginal, Operative vaginal, Pre-labour caesarean section, Caesarean section after onset of labour)
  - Indication for caesarean section
  - Method of anaesthesia (Regional/General)
- Did the woman go into labour?
  - If yes what was the onset of labour? (Spontaneous/Induced)

[If induced selected]

- Method of induction (Prostangladin/Artifical Rupture of Membrane/Oxytocin)
- Indication for induction (Fetal causes/Maternal causes/Other
- Was the woman given any pain control? Yes/No
  - If yes, Epidural analgesia/ Other
- Was antibiotic prophylaxis given during delivery? Yes/No
- Reason antibiotic prophylaxis not given (Patient declined/Other [Free text]
- Delivery complications
- Total blood loss at delivery
- Was a hysterectomy performed? Yes/No
- Was the woman admitted to ITU?
  - Complications (Postpartum haemorrhage/Infection/Other)

*Infant*

- Was the infant admitted to the neonatal unit? Yes/No
- Sex of infant
- 5 min Apgar
- Gestational age at birth (Extremely preterm [<28 wks]/Very preterm [28 to 32 wks]/Moderate to late preterm [32 to 37 wks]/Full term [>37 wks])
- Birthweight
- Was the infant still born? Yes/No

**Outcomes (To be completed no sooner than 28 days after delivery)**

*Woman*

- Did any major maternal morbidity occur? Y/N (If yes, Infection related/Pregnancy related/Leukaemia related/Other)
- Did the woman die? Y/N
  - If yes, date
- What was the primary cause of death as stated on the death certificate?
  - Infection/Bleeding/Leukaemia/others/not known
- Did the woman have any antenatal blood transfusions? (if yes date and units received)
- Transfusion Indication (Anaemia/Blood loss/Other)

*Infant*

- Did the infant have a congenital abnormality? (Y/N)
- If yes, please define (General; CNS; Musculoskeletal; Gastrointestinal; Genital/Urinary; Cardiovascular; Miscs
- Did any other major neonatal complications occur? Y/N
- Was an echocardiogram performed on the neonate? Y/N
  - If yes, Date of scan and Results (Heart failure/Heart enlargement (Cardiomegaly)/Normal)
- Did this infant die? Yes/No
  - If yes, Date of Death and cause

*Neonate full blood count*

- Date of test and details of full blood count results

**Follow-up eCRF (2 and 4 years post-delivery)**

- Has the woman died
  - If yes age at death
  - Primary cause of death (Infection/Bleeding/Leukaemia/Other)
- Has the woman experienced a relapse?
- Has the woman received a new diagnosis of any other acquired medical or psychological conditions?
- Has the woman had a cervical smear test during the two years?
  - If yes, date and result (Positive/Negative)
- Has the woman had any further pregnancies in the previous 2 years? Y/N
  - Number of pregnancies less than 24 weeks
  - Terminations [number box]

Number of pregnancies over 24 weeks (further pregnancies event)

- Has the MDS progressed to AML? (If yes, date of progression)

**Treatment eCRF**

*Start of treatment*

- ECOG Performance Score at start of treatment
- Height at treatment start
- Weight at treatment start

*Treatment*

- Induction therapy (Drug name, Date started, Daily dose, Route of administration, Number of Cycles, Date stopped, Cumulative Dose, Trimester)
- Consolidation therapy (Drug name, Date started, Daily dose, Route of administration, Number of Cycles, Date stopped, Cumulative Dose, Trimester)
- Any other treatment (Drug name, Date started, Daily dose, Route of administration, Number of Cycles, Date stopped, Cumulative Dose, Trimester)

*Other Treatment*

*APL patients only*

- Was ATRA administered during pregnancy? Y/N
- Date ATRA was started
- Indicate the treatment combination (ATRA alone until delivery/ATRA with idarubicin/alternative anthracycline until delivery/ATRA with idarubicin/alternative athracycline and cytarabine until delivery)
- If alternative anthracycline used, please specify

*MDS patients only*

- Date of progression

[If the patient received chemotherapy, please complete the Induction Therapy and Consolidation Therapy tables as per AL therapy on the Treatment tab.]

[If the patient received any other treatments (e.g. growth factors), please complete the Any Other Treatment Table as per AL therapy on the Treatment tab.]

- Was treatment given during pregnancy? Y/N (Should above notes be below this question opening with a ‘Yes’ answer?
- Has the MDS progressed to AML? (Y/N)

*Radiotherapy (Previous AL only)*

- Date started/Total dose/Number of fractions/Duration of Treatment
- Did the woman have gonadal shielding (Y/N)

*Side effects*

- Were there any reported side effects for the mother to any treatments?
- If yes report System organ class and grade (1-5) according to CTCAE V4.0
  - *Blood and lymphatic system disorders*
  - *Cardiac disorders*
  - *Congenital, familial and genetic disorders*
  - *Ear and labyrinth disorders*
  - *Endocrine disorders*
  - *Eye disorders*
  - *Gastrointestinal disorders*
  - *General disorders and administration site conditions*
  - *Hepatobiliary disorders*
  - *Immune system disorders*
  - *Infections and infestations*
  - *Injury, poisoning and procedural complications*
  - *Investigations*
  - *Metabolism and nutrition disorders*
  - *Musculoskeletal and connective tissue disorders*
  - *Neoplasms benign, malignant and unspecified (incl cysts and polyps)*
  - *Nervous system disorders*
  - *Pregnancy, puerperium and perinatal conditions*
  - *Psychiatric disorders*
  - *Renal and urinary disorders*
  - *Reproductive system and breast disorders*
  - *Respiratory, thoracic and mediastinal disorders*
  - *Skin and subcutaneous tissue disorders*
  - *Social circumstances*
  - *Surgical and medical procedures*
  - *Vascular disorders*

*Modifications*

- Were any modifications made to the treatment regimen as compared to the standard AML treatment regimen?
- Dose reduction (Y/N – If Yes, details)
- Time delay (Y/N – If Yes, details)
- Stopped early (Y/N – If Yes, details)

**Full blood count**

*Full blood count*

- Date of test
- Full blood count results

*APL/ALL subtype (Current AL Only)*

- WBC x10^9^/L (APL patients only: <10; >10
- WBC (B Cells) x10^9^/L (ALL patients only): <30; >30
- WBC (T Cells) x10^9^/L (ALL patients only): <100; >100

*Blood film*

- Was a blood film made? Y/N.
  - Dysplastic changes (Yes/No)
  - Result

**Biochemical Panel**

- Date of test
- Biochemical panel result

**Clotting data**

- Date of test
- Blood clotting results

**Bone marrow aspirate**

- Details of analyses performed

**Post treatment (Previous AL patients only)**

*Fertility*

- Was any form of fertility preservation carried out prior to treatment? (Y/N)
  - If yes, Embryo cryopreservation/Oocyte cryopreservation/Ovarian tissue cryopreservation/Other
- Was fertility assessed hormonally (Y/N)
  - If yes, Date of test/Result

*Outcomes*

- Did the woman experience amenorrhea after treatment? Y/N
  - If yes, date of menarche recovery
- Was the woman diagnosed with premature ovarian failure? (Y/N)
  - If yes, date of diagnosis
- Was the woman diagnosed with early menopause (Y/N)
  - If yes, date of diagnosis
- Was hormone replacement therapy (HRT) given after treatment? Y/N
  - If yes, date started
  - Ongoing (Y/N)
  - Duration (months)

*Follow-up*

- Last date patient seen
- Complete Remission Status
- ECOG Performance Status
