# Supplementary material for: A prospective registry-based cohort study of the diagnosis and management of acute leukaemia in pregnancy: Study protocol
Source: PLoS One. 2022 Feb 7;17(2):e0263195. doi: 10.1371/journal.pone.0263195 (PMC8820608; doi:10.1371/journal.pone.0263195)
Supplement: S1 Protocol — (PDF) [file pone.0263195.s002.pdf]

Leukaemia in Pregnancy Study (LIPS)
Synopsis

Full Protocol Title	***A Prospective registry-based cohort study to monitor the diagnosis and management of acute leukaemia in pregnancy.***
Sub Title	*Acute leukaemia in pregnancy cohort study.*
Protocol Version	*1.0*<br>*14th June 2019*
HHTU Ref	*P006*
IRAS ID	*238879*
REC	*19/CBS/3662*
Clinicaltrials.gov	*Tbc*
Study Design	*Registry based cohort study*
Study Parameters	*Diagnosis information*<br>*Pregnancy information*<br>*Delivery information*<br>*Follow-up information*
Authorised By	*Dr Sahra Ali*

A PROSPECTIVE REGISTRY-BASED COHORT STUDY TO MONITOR THE DIAGNOSIS AND MANAGEMENT OF ACUTE LEUKAEMIA IN PREGNANCY

Leukaemia **i**n **P**regnancy **S**tudy (LIPS)

1. Introduction 5
2. Aims and objectives 7
3. Data systems 8
4. Study Design 8
5. Selection and withdrawal of participants 9
6. Assessments and procedures 11
7. Statistics 13
8. Study supervision 13
9. Data handling and record keeping 13
10. Data access and quality assurance 14
11. Patient and Public Involvement 15
12. Publication 17
13. Finance 17
14. Regulatory Approval 17
15. References 17

ABBREVIATIONS

Definition of terms

AL	Acute leukaemia
AML	Acute myeloid leukaemia
ALL	Acute lymphoblastic leukaemia
ART	Assisted reproductive technology
ATRA	All-*trans*-retinoic acid
BCSH	British Committee for Standards in Haematology
COSD	Cancer Outcomes and Services Dataset
eCRF	Electronic case report form
HEY	Hull and East Yorkshire Hospitals NHS Trust
HHTU	Hull Health Trials Unit
HSCT	Haematopoietic stem cell transplantation
MDS	Myelodysplasia
POF	Premature ovarian failure
RCC	REDCap cloud
TMG	Trial Management Group
UKOSS	UK Obstetric Surveillance System
UOH	University of Hull

General information

Sponsor contact details
Dr David Richards
Pro Vice-Chancellor (Research and Enterprise)
University of Hull,
Hull
HU6 7RX
E-mail: pvc-re@hull.ac.uk
Telephone: 01482 456561

Data custodian contact details
Jeanette Strachan
University SIRO,
University of Hull,
Hull
HU6 7RX
E-mail: jeannette.strachan@hull.ac.uk
Telephone:01482 465302

Chief Investigator details
Dr Sahra Ali
Consultant Haematologist & Myeloid specialist
Departmental Research Lead
Speciality Lead for Cancer Division 1
NIHR/CRN HY
Queens Centre for Haematology & Oncology
Castle Hill Hospital, Hull
HU16 5JQ
E-mail: Sara.Ali@hey.nhs.uk
Telephone: 01482 461297

Local Principal Investigator
Dr David Allsup
Consultant Haematologist & Myeloid specialist
Departmental Research Lead
Speciality Lead for Cancer Division 1
NIHR/CRN HY
Queens Centre for Haematology & Oncology
Castle Hill Hospital, Hull
HU16 5JQ
Email: David.Allsup@hey.nhs.uk

Trials Unit contact details
Dr Judith Cohen
Director, Hull Health Trials Unit
3rd Floor, Allam Medical Building
University of Hull
HU6 7RX
E-mail: judith.cohen@hyms.ac.uk
Telephone: 01482 463382

Trial Manager
Dr Matthew Northgraves
Trial manager, Hull Health Trials Unit
3rd Floor, Allam Medical Building
University of Hull
HU6 7RX
E-mail: matthew.northgraves@hyms.ac.uk
Telephone: 01482 463373

Trial Management Group

CI, HHTU Trial Manager, HHTU Database Manager, HHTU Director, Statistician, Local PI

Protocol amendments since Version 1.0

None

STUDY SUMMARY

Acute leukaemia (AL) is an aggressive but potentially curable cancer that can affect women of childbearing age. When a pregnancy is complicated by a diagnosis of AL, clinicians face a complex dilemma: to balance risking the mother's survival through delaying treatment, against the potential harm to the foetus through exposure to cancer drugs. Reports suggest that, providing the first trimester is avoided, successful treatment of AL during pregnancy is possible, and considered safe. However, there is currently no standard approach to treatment of these women.

This observational study aims to monitor and record the current treatment and outcomes of patients diagnosed with acute leukaemia during or prior to pregnancy. Patients will receive the treatment recommended by their doctor, the study will not alter the treatment pathway of participants. This study will establish a new research database of Leukaemia in Pregnancy, initially collecting data from cases in the past ten years, and any new cases that are diagnosed during the current funding period.

The initial planned analyses from this dataset will enable more robust, evidence-based recommendations to be made on how to monitor and manage these patients, and will add value to and improve the existing British Committee for Standards in Haematology (BCSH) guidelines, which were largely derived from expert opinion. This should enable healthcare professionals to have greater confidence in managing these patients, leading to a more standardised approach to providing high quality care. The study will benefit NHS Trusts and patients across the UK through more informed clinical decision making with regards to the care they receive. It will also provide an important data resource which researchers can apply to use in further analyses, with plans to continue data collection if further funding is obtained.

1. INTRODUCTION

Acute leukaemia (AL is an aggressive, but potentially curable, neoplasm of haematopoietic origin, encompassing both acute myeloid leukaemia (AML; approximately two-thirds of cases) and acute lymphoblastic leukaemia (ALL) subtypes [1-3]. Both AML and ALL present in all ages, and can therefore affect women of childbearing age; AL may therefore be diagnosed during the course of a pregnancy, or a woman diagnosed and treated for AL may wish to have children at a later date.

Background

The true incidence of AL is pregnancy is not well known, but it is estimated to affect approximately 1 in 75,000 to 100,000 pregnancies [4]. Despite this rarity, AL is one of the most frequent malignant diseases observed during pregnancy [5]. When a pregnancy is complicated by a diagnosis of AL, this poses a unique clinical dilemma, with conflict between the risk of prognostic detriment incurred to the mother through delaying treatment and the potential for harm to the foetus as a result of exposure to chemotherapeutic drugs. Clinicians face very difficult decisions in planning the treatment of these patients; their management requires a specialised, cooperative multidisciplinary approach by consultant haematologists, obstetricians, anaesthetists and neonatologists.

The challenge of treating AL during pregnancy is greater than for solid tumours or lymphoma as its markedly aggressive nature necessitates higher doses of induction chemotherapy [6]. If left untreated, both mother and baby will likely die; however, evidence to date suggests that successful treatment of AL is both feasible and safe with the foetus *in utero* [7], with reported complete remission rates of up to 80% after standard chemotherapy [8, 9]. Furthermore, there are several reports of the successful use of all-*trans*-retinoic acid (ATRA) therapy in combination with conventional chemotherapy for the treatment of acute promyelocytic leukaemia (APL) [10-13]. Additionally, leukaemia treated to remission at the time of delivery has been associated with decreased maternal and foetal morbidity and mortality compared with untreated leukaemia [9].

The number of women who have previously been treated for AL but go on to become pregnant has not been studied. Accumulating evidence indicates that ovarian reserve can be adversely affected by chemotherapy in cancer patients [14], and the intensive cytotoxic therapy and sometimes craniospinal or total body irradiation required for the treatment of AL, can result in post-treatment premature ovarian failure (POF), with a high risk of induction of early menopause and infertility [15, 16]. Preconditioning regimens for haematopoietic stem cell transplantation (HSCT) comprising alkylating agents and/or irradiation carry a particularly high risk for germ cell injury, gonadal dysfunction and infertility [17] and, post-HSCT, these women face an increased risk of spontaneous abortions and miscarriages, pre-term delivery and low birth weight babies. Unfortunately, due to the aggressive nature of the disease, a lower intensity chemotherapy regimen cannot be considered in these women and, as treatment must be started immediately after diagnosis, their pre-treatment options to preserve fertility (e.g. embryo, oocyte or ovarian tissue cryopreservation)

are much reduced. However, fertility and parenthood remain important quality of life issues for cancer survivors of childbearing age [16] and so cannot be ignored.

Despite this, there have been reports of successful pregnancies following treatment of AML, APL and ALL, both with and without the use of assisted reproductive technology (ART) [18], and it appears that the likelihood of ovarian failure (and the chances of subsequent recovery of ovarian function) depend on a variety of factors including patient age, type of cytotoxic agent used, and the cumulative chemotherapy dose [15]. There is also a crucial need to gather data on the incidence of viable pregnancies among women who have previously received treatment for AL in order to better advise on the initial care of a young woman diagnosed with AL, as well as the management of any future pregnancies.

There is no standard approach for the treatment of AL during pregnancy and the effects on fertility post treatment are not fully known. This clinical dilemma also includes obstetric uncertainties such as mode and timing of delivery, use or not of enuraxial anaesthesia/general anaesthesia, optimisation of blood counts pre-delivery. It therefore remains an important need to collect data about the treatment and outcomes of these individuals. Current therapeutic approaches may be highly variable, and may differ from those used routinely to treat the disease in non-pregnant patients. Given the rarity of AL in pregnancy, there is a relative paucity of evidence concerning its diagnosis and management, currently limited to individual case reports, very small retrospective case series and reviews pertaining to only a small number of patients.

Performing large trials on these patients is virtually impossible, not only in practical terms because of the relatively small number of prospective cases, but also due to the ethical concerns around the potential exposure of foetuses to cytotoxic agents, and therefore few institutions are able to build sufficient clinical experience with this scenario.

The current British Committee for Standards in Haematology (BCSH) guidelines [19], specifically address and make recommendations on diagnosis and management issues associated with AML during pregnancy. However, although of great value, only low-quality evidence could be obtained to inform the guidelines and many of the recommendations were derived from expert opinion. Furthermore, for ALL scant available data at present regarding its treatment during pregnancy has so far precluded the establishment of firm recommendations [20]. With the prevalence of cancer during pregnancy expected to rise due to an increase in average age at pregnancy [21], there is a real need to consolidate firm recommendations on the effective therapeutic management of these patients.

The Swedish Acute Leukaemia Registry and Danish National Acute Leukaemia Registry both gather data on diagnosis, treatment response and survival [22, 23]; however, pregnancy in these patients is not studied in any great detail. For the NHS in England, the national standard for reporting cancer is the Cancer Outcomes and Services Dataset (COSD) [24], while the Systemic Anti-Cancer Therapy Dataset [25] collects data on the management of patients receiving cancer chemotherapy. However, these do not provide UK-wide coverage, and are also not designed to

capture the specifics relating to pregnancy. The UK Obstetric Surveillance System (UKOSS), established by the National Perinatal Epidemiology Unit at the University of Oxford, is a unique national reporting system for uncommon disorders of pregnancy [26], but does not include the required long-term patient follow-up in its studies and only collects prospective cases.

The purpose of this study is to capture both retrospective and prospective epidemiological, management and outcome data, to enable a more comprehensive assessment of the risks of cytotoxic therapy in this setting. The results will start to address the scarcity of evidence on the long-term effects of chemotherapy to a foetus exposed *in utero* and to a pregnancy conceived after completion of AL treatment. The study will serve a dual purpose: both as a supportive care study to monitor pregnancies during AL treatment, and also as a late effects study to report on the feasibility of viable pregnancies post-treatment. It will further provide a more accurate reflection of the management of AL during pregnancy, thereby allowing us to assess the impact of the introduction of the BCSH guidelines. It is intended to serve as a valuable resource to amalgamate and strengthen existing expertise on the topic, establishing a Leukaemia in Pregnancy research database. Initial planned analyses will inform more robust, conclusive, evidence-based patient management recommendations to better direct counselling and management of these patients, and to inform future patterns of care.

2. AIMS AND OBJECTIVES

This study aims to establish a research database to collect information about women who have a diagnosis of acute leukaemia (AL) or high-risk myelodysplasia (MDS) in pregnancy, or who have conceived after receiving treatment for either AL or high-risk MDS. Initial planned analyses will add to the evidence base, and the database will provide a source of data for future studies.

Primary outcome:

- To ascertain the outcomes of mothers and babies treated for AL or high-risk MDS during or prior to pregnancy.

Secondary outcomes:

- To document the current management practices for patients diagnosed with AL or high-risk MDS during pregnancy or who become pregnant after receiving therapy.
- To study the safety of any medication used and to identify toxicities to mothers and babies related to any chemotherapy given.
- To establish a Leukaemia in Pregnancy research database

3. DATA SYSTEMS

Data will be hosted and managed by the Hull Health Trials Unit (HHTU) using their secure online data capture system RedCap Cloud (RCC) and BOX Governance file storage system. HHTU hold a NHS Digital Data Security and Protection Toolkit covering these information systems.

Box is a cloud content management system which is ISO27001/ISO27018 certified and holds SOC1,2 and 3 reports. Box use IBM servers, the primary data centre is London with a backup in Frankfurt.

RCC is a cloud based EDC system provided by nPhase. Data is stored on dedicated RCC hardware in EU data centres (including real-time backup) managed by Amazon Web Services to industry standards outlined in ISO 27001, PCI DSS, SOC 1 - 3, FISMA, CIS, CSA, NIST and UK Cloud Security Principles. Data is encrypted at rest and in transit. RCC deliver compliance to HIPAA, CFR Part 11, and EMEA Annex 11.

Both of these systems have been through rigorous Information Governance and Security assessments during the University of Hull's procurement process.

4. STUDY DESIGN

A registry-based cohort study to identify and collect data on all women receiving a diagnosis of AL or high-risk myelodysplastic syndrome (MDS), and all women with a history of AL or high-risk MDS who fall pregnant within the study period. This is an observational study, and patients will receive usual care as determined by their Loco-regional multidisciplinary team.

In the UK, only hospitals with high intensity units treat AML and ALL, and therefore only these centres will be targeted for case identification. We aim to approach all consultant haematologists involved in the care of a woman diagnosed with AL during pregnancy, and request that they report all known cases, creating a national dataset.

For prospective cases, women will be identified at diagnosis and tracked throughout their pregnancy to allow comparison of the respective outcomes for both mother and neonate. There will be additional follow-up points at 2 and 4 years (as permitted within the duration of the study), to accurately document the subsequent outcomes of both mothers and neonates; 2 years is where the most incidences of relapse occur, and 4 years is considered decisive with regards to remission. In addition, consultant haematologists across the UK will be asked to provide retrospective data on any cases occurring since August 2009 (both pre- and post-introduction of the 2015 BCSH guidelines), to gain an overview of current and past approaches to patient management, and to serve as comparators to assess the current and future impact of the BCSH guidelines.

The methodology of data collection will be similar to that employed by the UK Obstetric Surveillance System (UKOSS) [27], which has received the approval of the London Multi-Centre Research

Ethics Committee (reference 04/MRE02/45). Within UKOSS, descriptive, case-control and anonymised cohort studies are conducted through a prospective, monthly case-collection scheme. Each month UKOSS sends electronic requests to nominated individuals at each Hospital site, inviting them to submit a case report. Where appropriate, more detailed data collection forms are then issued via post for completion.

We will use similar methodology to collect both retrospective and prospective data. Consultant haematologists will receive a reminder every 3 months via email. The email will include a link to the HHTU electronic data capture system where they will be able to submit any cases, either current or historical, into the study database. Once a case is identified, current patients or historical cases where the patient is still in active follow up or in regular contact with the clinical care team will be approached for consent. In historical cases where the patient is no longer in active follow-up or in regular contact with the clinical care team, cases will be processed without consent subject to the relevant approvals being gained.

Each patient entered onto the database will be designated a unique study identification number, generated automatically by the data capture system. Different case report forms will be activated accordingly as data is entered onto the system. Data will be entered directly into the database by the consultant haematologist through reference to medical records only, there will be no direct contact with patients. All data will be reported anonymously using the patient ID.

During the study set-up phase, consultant haematologists will be asked to provide an estimate of the number of cases encountered in the past ten years to enable a HHTU Trial Manager to monitor overall data collection rates. Email reminders will be issued, or the Local Clinical Research Team will be contacted by telephone, in order to minimise loss to follow-up. If a consultant haematologist is unwilling or too busy to provide a response, efforts will be made to identify an alternative suitable reporting individual at that site (e.g. an obstetrician) who would be part of the usual care team.

5. SELECTION AND WITHDRAWAL OF PARTICIPANTS

Inclusion

Women who have a diagnosis of acute leukaemia (AL) or high-risk myelodysplasia (MDS) in pregnancy, or who have later conceived after receiving previous treatment for either AL or high-risk MDS.

Exclusion

Pregnant women not meeting the inclusion criterion.

Consent

This study is an observational cohort, recording routinely collected data from patient clinical records only. It will not be practicable to obtain consent for data collection from all women due to the sensitivity of subject, as some women will no longer be in routine clinical care or may have died. To ensure the data collected is informative as possible, we need to aim for 100% collection.

Consent will be sought for:

Current cases

1. Where patients are currently receiving treatment and are **now** pregnant

   <u>Or</u>

2. Where patients have previously received treatment and are **now** pregnant.

Historical cases

1. Where patients had treatment whilst pregnant and are still in active follow up and/or contact with the clinical care team.

   <u>Or</u>

2. Where patients had a previous AL diagnosis and subsequently became pregnant post treatment that are still in active follow up and/or contact with the clinical care team.

For historical cases where the patient had treatment whilst pregnant or subsequently became pregnant post treatment but are now:

a) No longer in active clinical follow up. This may include: Discharged – either following treatment complete or as a result of non-attendance (DNA) of clinic appointments
b) No longer in contact with the clinical care team
c) Known to have died

Identifiable data will be collected without consent subject to the relevant approvals being given. Due to the sensitivity of the condition, contacting patients for consent if no longer in contact with the clinical care team could possibly cause unnecessary emotional distress to the patient and/or the family as some patients and/or their babies will not have survived.

Participants have no direct involvement in the study and will not be contacted directly beyond consent (if applicable). No site information or personal identifiable information will be contained within the analysis dataset. Data collection forms will be completed by the clinical research team who have permission to access their clinical data at the site as part of the usual care of the patient.

6. ASSESSMENTS AND PROCEDURES

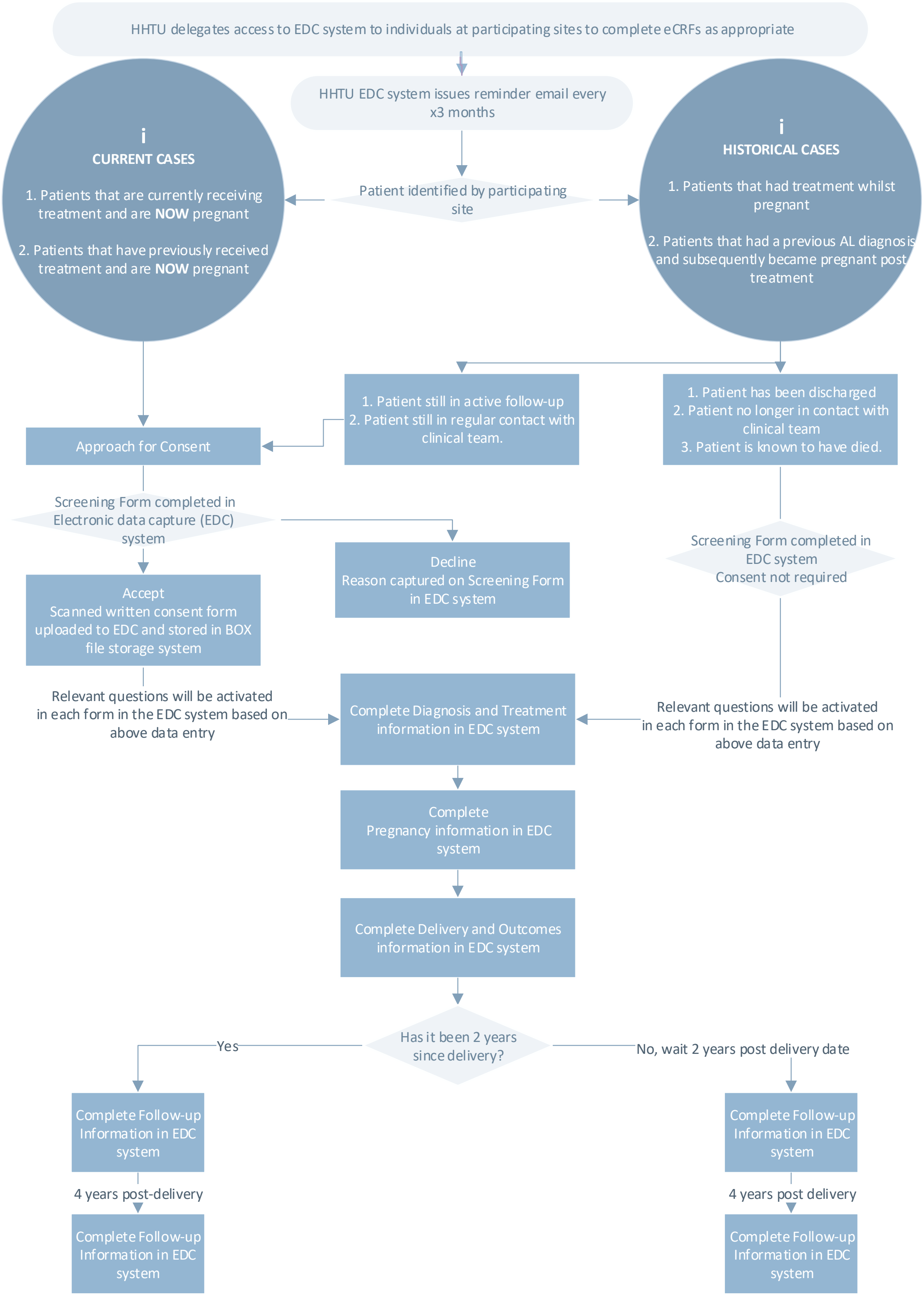

Once a case is identified, if the case is **current or the patient is still in active follow up or still in regular contact with the clinical care team,** the Consultant haematologist/designated person from the local clinical study team will provide the patient with the Participant Information Sheet, discuss the study and answer any questions the patient may have. Consent will be then be sought with the option for the patient to consent on the day. If the patient requests further time to consider we will arrange a more convenient time for them to come back and complete the consent process. If the case involves a **patient who has been discharged from active follow up, is no longer in contact with the clinical team or it is known the patient has died**, the data will be processed without consent providing they have not previously expressed a wish for their data to be used for the purposes of research. As HHTU will not be collecting any directly identifiable data, this will be done in accordance with each sites own local policy.

Once an eligible case is confirmed, the Consultant haematologist/designated person from the local clinical study team will log into RCC, HHTU's secure online database and complete the relevant electronic case report forms (eCRFs). A screening form will be completed for all, which will record the consent status (Yes/No/Not applicable). Depending on consent status ("Yes" or "not applicable"), the system will automatically generate a unique patient ID and more detailed eCRFs will be activated accordingly. For those not consenting, reason given for declining will be recorded on the screening CRF. When consent is required, scanned copies of the signed written informed consent form will be uploaded to RCC as part of the subject details eCRF. Further eCRFs will not be opened until the consent form is uploaded. RCC will partition the identifiable data within the system.

In order to maintain patient confidentiality, no addresses, postcodes, hospital or NHS numbers will be collected within the database. However, data will be linked in an anonymised format in order for participating sites to obtain further information from clinical records and to complete follow-up data collection forms at the appropriate time points. Individual sites contributing data will maintain a code sheet linking the patient study number to identifiable data, which they will store according to their local data protection policies.

More detailed eCRFs relate to the initial AL diagnosis for those patients diagnosed during pregnancy, or prior to pregnancy. Further eCRFs will then be completed at appropriate time points to allow data to be subsequently collected from each patient upon completion of therapy, after the expected delivery date (if not already captured within the 'completion of therapy' time-point) and then for 2 defined follow-up data collections. For patients with previous AL, pregnancy, delivery and follow-up information will be collected. The specific data to be collected will enable effective assessment of current management practices for these patients and will allow us to ascertain the rate of successful outcomes of both mothers and neonates, as well as to determine the safety and related toxicities of any chemotherapy administered.

7. STATISTICS

The statistical analysis will be mostly descriptive, and the resulting report will detail current and past management regimens and complications for AL in pregnancy, and will examine the impact of the introduction of the BCSH guidelines. Providing a satisfactory response rate is achieved, incidence data will be included, calculated with 95% confidence intervals. The LIPS Statistical Analysis Plan detailing the statistical analyses will be produced and signed off prior to the data analysis.

An interim report will be produced for funder review and research purpose. Final analysis will be carried out after the completion of data collection of all prospective cases. A summary report will be generated and circulated to all participating centres in order to evaluate the success of the study.

As the number of prospective cases expected is fairly low (approximately 10 cases per year), no strict sample size calculation has been made. The objective is to collect the maximum number of cases to improve the power of the study.

8. STUDY SUPERVISION

Whilst the chief investigator will have overall responsibility for the database, the day to day management of the database will be delegated to HHTU.

A trial management group (TMG) will meet regularly to discuss study implementation and progress. This will comprise of the Chief Investigator, Local Principal Investigator, HHTU Director, HHTU Study Manager, HHTU Information Systems Manager and the Statistician. A PPI representative will be invited to join the TMG. Reporting clinicians will be invited to join via teleconference at 6 monthly intervals. When possible, a face to face meeting including reporting clinicians will be arranged to coincide with national/meetings conferences.

The data collection will be conducted according to the International Conference on Harmonisation (ICH) Good Clinical Practice Guidelines. Meetings to discuss the data will be held on a quarterly basis. The group will provide six monthly reports of the progress, or completion, termination or discontinuation of the study to the main ethics committees and the Sponsor.

The study will also be highlighted regularly to all UK haematologists at both national and international meetings, prior to its launch and throughout the study period, with the aim of optimising recruitment rates, but also of informing on its progress.

9. DATA HANDLING AND RECORD KEEPING

The minimum amount of personal data possible will be collected for LIPS. For historical cases not requiring consent (e.g. patient is no longer in active follow up/in contact with the clinical care team), the only personal information obtained is year of birth for the mother, date of birth of child, date of death of mother/child (if applicable) and gender of child. This data will be held within the database

(RCC). For current cases and historical cases requiring consent, in addition, the patient name and signature will be captured as part of the consent process with scanned copies of the written informed consent uploaded into RCC. Consent forms will only be visible to designated staff at sites and the central HHTU team. HHTU will download and store a copy of the consent form in the BOX Governance file storage system.

Sites will be assigned a unique identifier for each patient for the purpose of data entry and monitoring. The usual care team at data collection sites will access the patient records and enter data into the database. Sites will hold a local log to enable them to go back to patient records and add subsequent data, which will be held according to local data protection policies. Any personal patient information obtained in the database during the course of the study will be anonymised prior to export of any dataset. No site information will be contained within the analysis dataset, therefore there will be no linkage back to the site.

All research staff working on LIPS will be required to work to the HHTU Confidentiality Code of Conduct, and adherence to this will be monitored according to the HHTU Confidentiality Audit Procedure. Data will be held in accordance with the General Data Protection Regulation (GDPR 2018). The study will be conducted in compliance with the current approved protocol, with Good Clinical Practice (GCP), and with applicable regulatory requirements.

Trial data will be collected and entered into the data capture system (RCC), with access restricted to approved and authorised users. Data is encrypted in transit and rest, sites can only view and enter data related to their own patients. It offers a robust audit log of data/time/user access and IP is maintained that shows all changes to data records within the system - all access in the system is tracked. Access reports will be reviewed at the TMG meetings.

10. DATA ACCESS AND QUALITY ASSURANCE

The database will be hosted and managed by the Hull Health Trials Unit using their secure online data capture system RCC and BOX Governance file storage system. Access to the EDC system is managed by HHTU staff who will create users on behalf of the study team. Users will be required to complete training and sign a terms of use document. Users are granted role based access which will be limited only to the sites they are working at. All activity within RCC is subject to full user audit including timestamp.

RCC restricts access to authorised study personnel and can assign individual access levels as appropriate using granular permissions. HHTU will ensure no direct patient identifiers are present in any exported datasets.

RCC audit reports will be included as a standing agenda item within the TMG meetings to ensure regular review. The study will be assigned a dedicated data manager from the HHTU to oversee and monitor the conduct of the database.

As part of the development process full specifications are generated for the study database listing each variable, eCRF and how they fit together into different time points (events) within the patient pathway. Development includes different environments for build, test and live, creating full validation records.

RCC supports automated checks on data entry appropriate to the question data type. These can either be: ‘hard’ checks where the form cannot be saved with the value entered or soft validations where the form can be saved but a query is raised which sites can respond to through the system. A combination of these checks will be specified, developed and tested as part of the controlled database development process.

RCC supports manual querying of data on the system. Queries are notified to site who will respond the through the system. This will be used by HHTU to raise appropriate queries on data that cannot be monitored through automated checks.

RCC supports labelling some fields as mandatory and others as optional. Where a field is mandatory the eCRF cannot be marked as complete and locked until there is data in it. These eCRFs will be chased by the HHTU as part of the ongoing data management process. The study dataset will be exported from RCC for analysis and stored in HHTU Box instance with restricted access. HHTU SOPs will be followed for the process of database lock and export including pseudo-anonymisation.

Following the planned completion of data collection and analysis funded by the Bloodwise grant, HHTU will develop and manage a process for request to access the data by other researchers. A data management panel, including if possible a PPI representative will be established to review and approve requests. There will be a requirement for researchers to read, agree and sign a data sharing agreement, HHTU acceptable use policies and SOPs prior to release of data. Anonymised data sets for release will be produced according to HHTU SOPs.

The study team will apply for further funding for maintenance and ongoing collection of data beyond the end of the current funding period.

11. PATIENT AND PUBLIC INVOLVEMENT

On the 11th June a PPI session was held with four members of the Trans-Humber Consumer Research Panel (2 males and 2 females) and a third female independent of the research panel. The panel included 2 males of retirement age and 2 females or 50 years of age. The final independent member was a third female aged in late 30s (mother of 3 with a previous experience of miscarriage). The director of HHTU and the Information systems manager of HHTU led the session. The first part of the session involved an overview of the study including the background and rationale for the study, what is a research database, how current and historical cases were being defined and why it was proposed not to seek consent for some cases. The panel were reminded that guidance states that consent should be taken if ‘reasonable and practicable’ and

then asked if *'Do you think we have considered this appropriately for both current and historical cases?'* and *'Do you think it is acceptable to process this data without consent in these circumstances?'*. The panel agreed that care and consideration had be given to the proposal and felt that it was acceptable not to take consent in the situations defined and agreed with the reasons explained. The female participants in the session suggested that in some circumstances knowing that the research is taking place may actually provide comfort to the individuals and their families and therefore, it would be important to disseminate the fact the research is taking place in a publicly accessible way. The panel emphasized the need for a clear pathway for the identification and possible removal of data, and that this should be included in the Privacy notice on the study website.

The next part of the session explained the process for taking consent from patients who were current cases or historical cases still in active follow-up. The panel were then asked *'Do you think it is reasonable to request consent on the same day?'* and *'Is it acceptable for the consultant to discuss the study and take consent?'.* It was agreed that it would be reasonable to take consent on the day if the patient agreed as there is no further patient involvement beyond giving informed consent. Equally, patients should be allowed to consent at a future appointment or arrange an alternative appointment if they preferred to have more time to think about it. The female members of the panel in particular felt that having the conversation with the consultant would be more reassuring that than with an independent member of staff.

During the third part of the session, the electronic data capture system to be used on LIPS, including the granular permission capability of RCC, and data protection and security procedures were explained to the panel by the HHTU Information systems manager. The panel expressed no concerns about the use of these systems and the procedures in place. The sharing of data for use in future research was then discussed, including possible ways of reducing identifiers in exported datasets and plans to form a data management panel to review access requests. The panel were then asked *'Do you think PPI representation on the data release governance panel is a good idea?'.* The panel were happy with the possible methods explained to further make the data less identifiable in shared exported datasets and were in agreement that PPI representation would be beneficial in the data release governance structure. In particular, it was suggest this would give a patient perspective on whether it was a legitimate use of the data. One panel member suggested as possible future end users of the database, input could be sought from obstetricians or gynaecologists regarding intended data items.

Finally, the panel were asked *'Do you think the study information and data handling is reflected in the PIS?', 'Is there anything missing or unclear?'* and *'Also for the consent form –any comments on the content?.* Apart from a couple of minor edits (e.g. addition of contact details to PIS, making clear in the consent form patients were consenting to the use of their data), the panel were happy with the content.

12. PUBLICATION

Researchers will follow open access guidelines when publishing the data, targeting high quality journals with good visibility. Additionally, where copyright allows, articles will be made available on research community websites such as Research Gate to enable wider dissemination.

The overall results of the study will be made publically available on ClinicalTrials.gov, via the HHTU project webpage and via the Bloodwise Cancer Charity news outlets and other dissemination routes.

The TMG will establish the authorship policy. Clinicians who contribute at least one case and fulfil in ICMJE (International Committee of Medical Journal Editors) recommendations will be offered authorship on the main results paper.

13. FINANCE

This study is funded via a Project Grant awarded by Bloodwise (The blood cancer research charity). Project Grant Ref 17016.

14. REGULATORY APPROVAL

This study has approval from X REC, Ref no xx.

Approvals for the use of historical personal data without consent

Confidentiality Advice Group (England and Wales)	Date:
Patient Benefit and Privacy Panel (Scotland)	Date:
Privacy Advisory Committee (Northern Ireland)	Date:

All regulatory approvals required will be submitted in line with HHTU standard operating procedures (SOPs).

15. REFERENCES

1) O'Donnell, M.R., Abboud, C.N., Altman, J., Appelbaum, F.R., Arber, D.A., Attar, E., Borate, U., Coutre, S.E., Damon, L.E., Goorha, S., Lancet, J., Maness, L.J., Marcucci, G., Millenson, M.M., Moore, J.O.,Ravandi, F., Shami, P.J., Smith, B.D., Stone, R.M., Strickland, S.A., Tallman, M.S., Wang, E.S., Naganuma, M., and Gregory, K.M., *Acute Myeloid Leukemia.* Journal of the National Comprehensive Cancer Network, 2012. **10**(8): 984-1021.

2) Hurley, T.J., McKinnell, J.V., and Irani, M.S., *Hematologic malignancies in pregnancy.* Obstet Gynecol Clin North Am, 2005. **32**(4): 595-614.

3) Khandaker, S. and Munshi, S., *A Rare Case of Acute Lymphoblastic Leukaemia in Pregnancy-Unique Maternal-Fetal Challenges.* Journal of Clinical and Diagnostic Research : JCDR, 2014. **8**(10): OD10-OD12.

4) Pavlidis, N.A., *Coexistence of Pregnancy and Malignancy.* The Oncologist, 2002. **7**(4): 279 287.

5) Menezes, J., Emerenciano, M., Pimenta, F., Filho, G.G., Magalhães, I.Q., Sant'Ana, M., Vaskuez, M.L., Renault, I.Z., and Pombo-de-Oliveira, M.S., *Occurrence of Acute Myeloid Leukemia in Young Pregnant Women.* Clinical Medicine Insights: Blood Disorders, 2008. **1**(CMBD-1-Emerenciano-et-al).

6) Ticku, J., Oberoi, S., Friend, S., Busowski, J., Langenstroer, M., and Baidas, S., *Acute lymphoblastic leukemia in pregnancy: a case report with literature review.* Therapeutic Advances in Hematology, 2013. **4**(5): 313-319.

7) Chelghoum, Y., Vey, N., Raffoux, E., Huguet, F., Pigneux, A., Witz, B., Pautas, C., de Botton, S., Guyotat, D., Lioure, B., Fegueux, N., Garban, F., Saad, H., and Thomas, X., *Acute leukemia during pregnancy: a report on 37 patients and a review of the literature.* Cancer, 2005. **104**(1): 110-117.

8) Döhner, H., Estey, E.H., Amadori, S., Appelbaum, F.R., Büchner, T., Burnett, A.K., Dombret, H., Fenaux, P., Grimwade, D., Larson, R.A., Lo-Coco, F., Naoe, T., Niederwieser, D., Ossenkoppele, G.J., Sanz, M.A., Sierra, J., Tallman, M.S., Löwenberg, B., and Bloomfield, C.D., *Diagnosis and management of acute myeloid leukemia in adults: recommendations from an international expert panel, on behalf of the European LeukemiaNet.* Blood, 2010. **115**(3): 453 474.

9) Reynoso, E.E., Shepherd, F.A., Messner, H.A., Farquharson, H.A., Garvey, M.B., and Baker, M.A., *Acute leukemia during pregnancy: the Toronto Leukemia Study Group experience with long-term follow-up of children exposed in utero to chemotherapeutic agents.* Journal of Clinical Oncology, 1987. **5**(7): 1098-1106

10) Carradice, D., Austin, N., Bayston, K., and Ganly, P.S., *Successful treatment of acute promyelocytic leukaemia during pregnancy.* Clin Lab Haematol, 2002. **24**(5): 307-11.

11) Ganzitti, L., Fachechi, G., Driul, L., and Marchesoni, D., *Acute promyelocytic leukemia during pregnancy.* Fertil Steril, 2010. **94**(6): 2330 e5-6.

12) Agarwal, K., Patel, M., and Agarwal, V., *A Complicated Case of Acute Promyelocytic Leukemia in the Second Trimester of Pregnancy Successfully Treated with All-trans-Retinoic Acid.* Case Rep Hematol, 2015.**2015**: 634252.

13) Sanz, M.A., Montesinos, P., Casale, M.F., Diaz-Mediavilla, J., Jimenez, S., Fernandez, I., Fernandez, P., Gonzalez-Campos, J., Gonzalez, J.D., Herrera, P., de Lisa, E., Olave, T., Rojas, R., Salamero, O., Sayas, M.J., Pellicer, A., and Perales, A., *Maternal and fetal outcomes in pregnant women with acute promyelocytic leukemia.* Ann Hematol, 2015. **94**(8): 1357-61.

14) Das, M., Shehata, F., Son, W.Y., Tulandi, T., and Holzer, H., *Ovarian reserve and response to IVF and in vitro maturation treatment following chemotherapy.* Hum Reprod, 2012. **27**(8): 2509 14.

15) Naessen, S., Bergstrom, I., Ljungman, P., and Landgren, B.M., *Long-term follow-up of bone density, general and reproductive health in female survivors after treatment for haematological malignancies.* Eur J Haematol, 2014. **93**(2): 137-42.

16) Stensheim, H., Cvancarova, M., Moller, B., and Fossa, S.D., *Pregnancy after adolescent and adult cancer: a population-based matched cohort study.* Int J Cancer, 2011. **129**(5): 1225-36.

17) Carter, A., Robison, L.L., Francisco, L., Smith, D., Grant, M., Baker, K.S., Gurney, J.G., McGlave, P.B., Weisdorf, D.J., Forman, S.J., and Bhatia, S., *Prevalence of conception and pregnancy outcomes after hematopoietic cell transplantation: report from the Bone Marrow Transplant Survivor Study.* Bone Marrow Transplant, 2006. **37**(11): 1023-9.

18) Wang, W.S., Tzeng, C.H., Hsieh, R.K., Chiou, T.J., Liu, J.H., Yen, C.C., and Chen, P.M., *Successful pregnancy following very high-dose total body irradiation (1575 cGy) and bone marrow transplantation in a woman with acute myeloid leukemia.* Bone Marrow Transplant, 1998. **21**(4): 415-7.

19) Ali, S., Jones, G.L., Culligan, D.J., Marsden, P.J., Russell, N., Embleton, N.D., Craddock, C., and the British Committee for Standards in, H., *Guidelines for the diagnosis and management of acute myeloid leukaemia in pregnancy.* British Journal of Haematology, 2015. **170**(4): 487-495.

20) Shapira, T., Pereg, D., and Lishner, M., *How I treat acute and chronic leukemia in pregnancy.* Blood Reviews, 2008. **22**(5): 247-259.

21) Brenner, B., Avivi, I., and Lishner, M., *Haematological cancers in pregnancy.* The Lancet, 2012. **379**(9815): 580-587.

22) Juliusson, G., Lazarevic, V., Hörstedt, A.-S., Hagberg, O., and Höglund, M., *Acute myeloid leukemia in the real world: why population-based registries are needed.* Blood, 2012. **119**(17): 3890-3899.

23) Ostgard, L.S., Norgaard, J.M., Severinsen, M.T., Sengelov, H., Friis, L., Jensen, M.K., Nielsen, O.J., and Norgaard, M., *Data quality in the Danish National Acute Leukemia Registry: a hematological data resource.* Clin Epidemiol, 2013. **5**: 335-344.

24) National Cancer Intelligence Network. Cancer Outcomes and Services Dataset (COSD). Available from http://www.ncin.org.uk/collecting_and_using_data/data_collection/cosd.

25) National Cancer Intelligence Network. SACT Systemic Anti-Cancer Therapy Dataset website. Available from http://www.chemodataset.nhs.uk/home.

26) National Perinatal Epidemiology Unit (NPEU), University of Oxford. UK Obstetric Surveillance System (UKOSS). Available from https://www.npeu.ox.ac.uk/ukoss.

27) National Perinatal Epidemiology Unit (NPEU), University of Oxford. UK Obstetric Surveillance System (UKOSS) Methodology. Available from https://www.npeu.ox.ac.uk/ukoss/methodology.
